# GWRA: grey wolf based reconstruction algorithm for compressive sensing signals

**DOI:** 10.7717/peerj-cs.217

**Published:** 2019-09-02

**Authors:** Ahmed Aziz, Karan Singh, Ahmed Elsawy, Walid Osamy, Ahmed M. Khedr

**Affiliations:** 1 Computer Science Department, Faculty of Computers and Artificial Intelligence, Benha University, Benha, Egypt; 2 School of Computer and Systems Sciences, Jawaharlal Nehru University, New Delhi, Delhi, India; 3 Department of Computer Science, University of Sharjah, Sharjah, UAE, United Arab Emirates

**Keywords:** Average normalized mean squared error, Compressive sensing, Greedy-based reconstruction algorithm, Grey wolf optimizer, Mean absolute percentage error, Reconstruction algorithms

## Abstract

The recent advances in compressive sensing (CS) based solutions make it a promising technique for signal acquisition, image processing and other types of data compression needs. In CS, the most challenging problem is to design an accurate and efficient algorithm for reconstructing the original data. Greedy-based reconstruction algorithms proved themselves as a good solution to this problem because of their fast implementation and low complex computations. In this paper, we propose a new optimization algorithm called grey wolf reconstruction algorithm (GWRA). GWRA is inspired from the benefits of integrating both the reversible greedy algorithm and the grey wolf optimizer algorithm. The effectiveness of GWRA technique is demonstrated and validated through rigorous simulations. The simulation results show that GWRA significantly exceeds the greedy-based reconstruction algorithms such as sum product, orthogonal matching pursuit, compressive sampling matching pursuit and filtered back projection and swarm based techniques such as BA and PSO in terms of reducing the reconstruction error, the mean absolute percentage error and the average normalized mean squared error.

## Introduction

Exploiting the sparse nature of the signals is highly challenging in various signal processing applications such as signal compression, inverse problems and this motivated the development of compressive sensing (CS) methodologies (Donoho, 2006). CS provides an alternative new method of compressing data, offering a new signal sampling theory which we can adopt in variety of applications including data and sensor networks (Cevher & Jafarpour, 2010), medical systems, image processing and video camera, signal detection, analog-to-digital convertors (Choi et al., 2010) and several other applications.

The CS reconstruction problems are solved by convex algorithms and greedy algorithms (GAs). Convex algorithms are not efficient because they require high complex computations. Thus, most of researchers choose GAs, which are faster and give the same performance as convex algorithms in terms of minimum reconstruction error. On the other hand, GAs do not give a global solution as all heuristics algorithms that execute blind search and usually stuck on local optima. In this paper, we use grey wolf optimizer (GWO), which is considered as a meta heuristic algorithm that is prominent in finding global solution. Only a few works involving swarm algorithms have been proposed to solve CS reconstruction problem such as in Bao et al. (2018) and Du, Cheng & Liu (2013) where the authors used BAT and PSO algorithms to reconstruct the CS data. However, these two algorithms (Bao et al., 2018; Du, Cheng & Liu, 2013) have a number of drawbacks such as slow convergence velocity and tend to fall in local optimum status easily. In contrast, the GWO algorithm showed better performance than other swarm optimization algorithms (Mirjalili, Mohammad Mirjalili & Lewis, 2014).

## Problem Formulation

Consider *x*[*n*], where *n* = 1, 2 … *N*, denotes sensor nodes reading vector set, *N* represents the count of sensor nodes. Any individual signal in *R^N^* can be expressed using basis of *N* × 1 vectors {Ψ_*i*_}_*i*=1_^*N*^. Employing the basis *N* × *N* matrix, expressed as =[Ψ_1_|Ψ_2_|Ψ_3_|....|Ψ_*N*_], together with the vectors Ψ_*i*_ being the columns, we can represent the signal *x* as given below (Donoho, 2006):

(1) $$x = \sum\limits_{i = 1}^N {{g_i}\,{\Psi _i}} .$$

This representation is done in terms of *N* × *N* orthonormal basis transform matrix. Here, *g* denotes the *N* × 1 sparse presentation of *x*. CS focuses on signals with a sparse representation. The number of basis vectors of *x* is *S*, such that *S* << *N*. Also we have, (*N* − *S*) values of *g* are zeros and only *S* values are non-zeros. Using Eq. (1), the compressed samples *y* (compressive measurements) can be obtained as:

(2) $$y = {\rm\phi} {\rm{}}x = {\rm\phi} {{\Psi }}g = {\rm{\theta }}g.$$

Here, the compressed samples vector *y* ∈ *R^M^*, with *M* << *N* and θ is *M* × *N* matrix.

The challenge of solving an undetermined set of linear equations have motivated the researchers to investigate upon this problem and as a result, diverse practical applications emerged to meet this challenge. In CS approach, the main responsibility is to offer an efficient reconstruction method enabling the recovery of the large and sparse signal with the help of a few available measurement coefficients. The reconstruction of signal using this available incomplete set of measurements is really challenging and relies on the sparse representation of signal. An easiest approach for recovering the original inherent sparse signal using its small set of linear measurements as shown in Eq. (2) is to compute the number of non-zero entries obtained by solving **‖*L*‖_0_** minimization problem. The reconstruction problem can thus be expressed as

(3) $$x = \arg \;\min {\left\| x \right\|_0}\quad {\rm{subject \,to\quad \,}}y = {\rm\phi} x$$

The ‖*L*‖_0_ minimization problem works well in theoretical aspects, but in general, it is an NP-hard problem (Mallat & Wavelet, 1999; Candes & Tao, 2006) and hence Eq. (3) is computationally intractable for any vector or matrix.

The main task involved in CS is to reconstruct the compressed sparsely sampled signal, involving solutions to an undetermined set of linear equations, with undefined set of solutions. Therefore, an efficient reconstruction algorithm is required to recover the inherent sparse signal. Main aim of signal reconstruction procedure is to evaluate the possible solutions derived from the inverse equation defined above so that it is possible to find the most appropriate estimate of the original sparse signal. The original signal reconstruction problem can be viewed as an optimization problem and numerous algorithms have been proposed with this intention. According to the CS method, the reconstruction algorithms for recovering the original sparse signal can be broadly categorized into two types: (i) convex relaxation, (ii) GA. Convex relaxation based optimization corresponds to a class of algorithms which make use of linear programming approach to solve the reconstruction problem. These techniques are capable of finding optimal/near optimal solutions to the reconstruction issues, but they have relatively high computational complexity. The examples for such algorithms are least absolute shrinkage and selection operator, basis pursuit and basis pursuit de-noising. In order to overcome the computational complexity of recovering the sparse signal, a family of GA/iterative algorithms have been introduced. GA solves the reconstruction problem in greedy/iterative fashion, with reduced complexity (Chartrand & Yin 2008). Therefore, GA is more adoptable for signal reconstruction in CS. GA techniques are classified into two categories: (i) reversible, (ii) irreversible. Both of them follows identical steps, detects the support-set making use of matched filter (MF) and after that constructs the original sparse signal using least squares (LS) method. In reversible GA, an element inserted to the support-set can be removed anytime, following a backward step. However, in irreversible GA, an element inserted to the support-set will remain there until the search ends. Examples for reversible GA includes sum product (SP; Dai & Milenkovic, 2009), compressive sampling matching pursuit (CoSaMP; Needell & Tropp, 2009) etc., whereas orthogonal matching pursuit (OMP; Tropp & Gilbert, 2007) belongs to the class of irreversible GA algorithms.

The authors of Mirjalili, Mohammad Mirjalili & Lewis (2014) proposed a swarm intelligent technique, GWO, well tested with 29 benchmark functions. The benchmark functions used are minimization functions and are divided into four groups: unimodal, multimodal, fixed-dimension multimodal and composite functions. The GWO algorithm is compared to PSO as an SI-based technique and GSA as a physics-based algorithm. In addition, the GWO algorithm is compared with three EAs: DE, fast evolutionary programing and evolution strategy with covariance matrix adaptation. The results showed that GWO is able to provide highly competitive results compared to well-known heuristics such as PSO, GSA, DE, EP and ES. First, the results on the unimodal functions showed the superior exploitation of the GWO algorithm. Second, the exploration ability of GWO is confirmed by the results on multimodal functions. Third, the results of the composite functions showed high local optima avoidance. Finally, the convergence analysis of GWO confirmed the convergence of this algorithm. Finding the global optimum precisely requires balancing the exploration and exploitation (i.e., good equilibrium) and this balance can be achieved using GWO (Faris et al., 2018).

Here, we propose a new grey wolf based reconstruction algorithm (GWRA) for CS signal reconstruction. GWRA algorithm is inspired from the GWO and the reversible GA. GWRA has two forward steps (*GA forward* and *GWO forward*) and one backward step. During the first iteration, GWRA matches filter detection to initialize the support set (*GA forward step*) and adds *q* elements to it. Then, GWRA increases the search space in this iteration by selecting extra *K* elements depending on GWO algorithm (*GWO forward step*) and then solves the LS equation to select the best *k* elements from *q* + *K* elements (*backward step)*.

Summary of the contributions in this paper:

1. Develop a novel reconstruction algorithm based on grey wolf optimizer (GWRA) that: (a) utilizes the advantages of GAs to initialize the forward steps and (b) utilizes the advantages of GWO algorithm that enlarges the search space to determine the optimal output and recover the data.
2. Provide extensive experiments, and the subsequent results illustrate that GWRA exhibit high performance results than the existing techniques in terms of reconstruction error.

The rest of this paper is divided as follows: the related research of the proposed problem is described in the section “Related Research.” In the section “Grey Wolf Optimizer Background” presents the GWO background. Then in section “Grey Wolf Reconstruction Based Algorithm,” we introduce our method to solve the proposed problem with the illustration of a numerical example scenario. The simulation results of our approach and a case study scenario is given in the section “Simulation Results.” Finally, the paper is concluded in the section “Conclusion.” Table 1 explains the abbreviations which are used this manuscript. Table 2 shows the notations used throughout the paper.

**Table 1:** The following abbreviations are used in this manuscript.

CS	Compressive sensing
IoT	Internet of things
MAPE	Mean absolute percentage error
GA	Greedy algorithm
ANMSE	Average normalized mean squared error
CoSaMP	Compressive sampling matching pursuit
OMP	Orthogonal matching pursuit algorithm
GWO	Grey wolf optimizer
MP	Matching pursuit
FBP	Filtered back projection
SP	Sum-product algorithm
BP	Basis pursuit

**Table 2:** Table of notations.

Notation	Description
*x*	Original signal
*M*	Number of measurements
*y*	Compressed sample
ϕ	CS matrix
Ψ	Transform matrix
*K*	Signal sparsity level
*g*	Sparse presentation of *x*
*r*	Residual of *y*
*X*	Wolf position
*X_p_*	Prey position
*q*	Number of selected columns by Wolf algorithm
*X*_α_	α Wolf position
*X*_β_	β Wolf position
*X*_δ_	δ Wolf position
*R*	Support set
*C*	Search set
φ_*c*_	Sub-matrix contains columns with indices *c* from φ matrix
best	Best solution or *X*_α_
Sec_best_	Second best solution or *X*_β_
thir_best_	Third best solution or *X*_δ_
*f*	Fitness value
*x*′	Estimated solution
*t*	Number of iterations
†	Pseudo-inverse
*L*	Indices set of largest *K* magnitude entries in ${{\varphi} _c}^\dagger y$

## Related Research

Compressive sensing has become an attractive approach, convenient for use in internet of things (IoT) platforms, which utilizes the sparse nature of sensor signals. The signal is compressed (reduce signal dimension) from *N* to *M* such that *M* << *N*, which will result in transmission of fewer samples, making it suitable for IoT applications that hold continuous data. The main challenge in CS approach is to provide reconstructed signal with an acceptable quality. Several reconstruction algorithms have been developed to meet this requirement. The convex reconstruction approach converts the problem defined in Eq. (3) to convex optimization problem, replacing non-convex *L*_0_ minimization problem with convex *L*_1_, as defined in Eq. (4).

(4) $$x = \arg \;\min {\left\| x \right\|_1}\quad {\rm{subject \,to\quad \,}}y = {\rm\phi} x$$

Equation (4) is then solved using the *L*_1_-magic toolbox (Davenport et al., 2010) or any such problem solvers or using any linear programming methods. Although these techniques are capable of finding optimal/near optimal solutions to the reconstruction issues, the relatively high computational complexity make them inappropriate for IoT applications.

On the other hand, GA-based algorithms could be suitable for IoT networks, as they solve the reconstruction problem with low computation and reduced complexity. In Mallat & Zhang (1993) matching pursuit algorithm (MP) is considered as the first GA based algorithm in which the support-set is initialized by the index of the largest magnitude entries in φ^T^*y*, this step is called *forward step* and then it solves the LS problem. However, MP algorithm does not consider the non-orthogonality of the CS matrix which leads to incorrect selection to the columns corresponding to the non-zero values. This drawback has been solved by OMP algorithm (Tropp & Gilbert, 2007). The OMP algorithm selects the index of the largest magnitude entries in φ^T^*r* during each iteration, where *r* is the residual of *y*, and then solves the LS problem. Different algorithms have been proposed based on OMP algorithm as in Donoho et al. (2012) and Needell & Vershynin (2009). In Donoho et al. (2012), a faster and enhanced version of OMP called stagewise OMP (StOMP) is proposed. StOMP enhances the forward step of OMP by selecting a number of columns, instead of one column as in OMP, the magnitude values of the columns in φ^T^*r* are greater than a threshold and then uses these columns for solving the LS problem. In Needell & Vershynin (2009), in each iteration, the inner-products with similar magnitudes are grouped into sets and the maximum energy set is then selected.

The above algorithms are classified as irreversible GA class, as they do not have a *backward step. Backward step* allows the algorithm to remove the wrong selection of elements during the *forward step*, i.e., in these algorithms, once an element is inserted to the support-set this element remains there until the search ends.

However, in reversible GAs such as SP (Dai & Milenkovic, 2009), IHT (Cevher & Jafarpour, 2010), CoSaMP (Needell & Tropp, 2009) and filtered back projection (FBP; Burak & Erdogan, 2013) algorithms, *backward step* is used to prune the wrong elements that have been added to the support-set during the *forward step*.

In CoSaMP and SP, initialization of support-set is done by placing the indices of *b* largest-magnitude components of Φ′*y*. The size of *b* is different in each algorithm, for example, *b* = *K* in SP and *b* = 2*K* in CoSaMP where the value of sparsity level *K* is known. On the other hand, FBP (Burak & Erdogan, 2013) algorithm has the ability to perform without the knowledge of *K*. It assigns forward and backward step size depending on the measurements size. In Cevher & Jafarpour (2010), the IHT algorithm considers iterative gradient search algorithm which updates the estimate-set depending on *e* gradient of the residue and keeps only the largest *K* entries by removing the wrong selection.

Even though GA based reconstruction have become significantly popular for recovery of CS signals, in general they do not provide optimal solution to the problem of CS reconstruction (Du, Cheng & Chen, 2014).

In Bao et al. (2018), the authors utilized the efficiency of the swarm algorithm BAT in finding the optimal solution of CS reconstruction problem. Also, in Du, Cheng & Liu (2013), PSO algorithm is used for CS data reconstruction. The results showed that GWO is able to provide highly competitive results compared to well-known heuristics algorithms such as PSO, GSA, DE, EP and ES (Mirjalili, Mohammad Mirjalili & Lewis, 2014). In contrast, the GWO algorithm displays better performance than other swarm optimization algorithms. Here, we introduce a new technique (GWRA), integrating the advantages of both GA and GWO in determining the optimal output for the desired problem of CS reconstruction.

## Grey Wolf Optimizer Background

**Figure 1:**
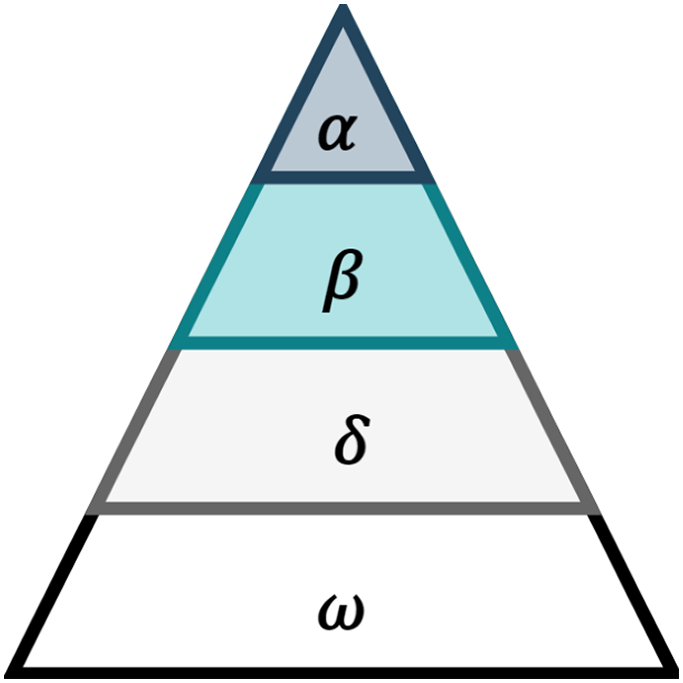
Grey wolfs’ hierarchal leadership (Faris et al., 2018).

Grey wolf optimizer can be defined as an intelligent meta-heuristic approach, inspired by group hunting behavior of grey wolves (Mirjalili, Mohammad Mirjalili & Lewis, 2014). The GWO method simulates the social behavior and hierarchy of grey wolves and their hunting method. The hierarchal leadership divides the grey wolves into four categories: (i) alpha (α), (ii) beta (β), (iii) delta (δ) and (iv) omega (ω) as shown in Fig. 1.

The α grey wolves are principally the leaders of their strict dominant hierarchy, responsible and powerful for decision making and leads the whole group during hunting, feeding, migration etc. The subordinates of alpha wolves are called β wolves and they are placed on the second level of the grey wolves’ hierarchy. They act as advisors and help the alpha wolves in making decisions. Finally, δ wolves execute alpha and beta wolves’ decision and manage ω wolves which are considered as the lowest ranking members of grey wolves hierarchy.

In GWO, α, β and δ guide the optimization process, where GWO considers the best solution and position for α wolves. In addition, the second and third best solutions and positions are assigned for β and δ, respectively. The other solutions are called ω solutions which always follow the solution of the other three wolves.

The mathematical representation of surrounding the prey and hunting process in GWO algorithm can be modelled as follows:

### Surrounding the prey

In the hunting process, the first step of grey wolves is “surrounding the prey,” which can be expressed mathematically as:

(5) $$D = \left| {C{X_p} - x\left( t \right)} \right|$$

(6) $$X\left( {t + 1} \right) = {X_p} - AD$$

Equation (5) expresses the distance between the wolf and the prey, where *X* is the wolf position, *X_p_* is the prey position, *t* denotes the current iteration and *C* is coefficient vector which can be calculated using Eq. (7). The wolf’s position is updated using Eq. (6), where *A* denotes the coefficient vector and it can be calculated using Eq. (8).

(7) $$C = 2{r_2}$$

(8) $$A = 2a{r_1}-a$$

Here, *r*_1_ and *r*_2_ are random values in [0, 1] and the values of *a*’s linearly decrease from 2 to 0 in each iteration.

### GWO hunting process

After surrounding prey process, α, β and δ wolves lead the hunting process. During the hunting process, GWO preserves the first three best solutions (according to their fitness values) for α, β and δ, respectively and according to the position of wolves α, β and δ, the other search agents (ω) estimates their positions. Then, they start to attack the prey. The behavior of this process can be represented mathematically as in Eqs. (9–11) (Faris et al., 2018):

(9) $${D_{\rm{\alpha }}} = \left| {{C_1}{X_{\rm{\alpha }}} - X} \right|,{D_{\rm{\beta }}} = \left| {{C_2}{X_{\rm{\beta }}} - X} \right|,{D_{\rm{\delta }}} = \left| {{C_3}{X_{\rm{\delta }}} - X} \right|$$

(10) $${X_1} = {X_{\rm{\alpha }}} - {A_1}{D_{\rm{\alpha }}},\ {X_2} = {X_{\rm{\beta }}} - {A_2}{D_{\rm{\beta }}},\ {X_{\rm{\delta }}} = {X_{\rm{\delta }}} - {A_3}{D_{\rm{\delta }}}$$

(11) $$X\left({t + 1} \right) = {{{X_1} + {X_2} + {X_3}} \over 3}$$

After updating the positions of all wolves, the hunting process starts the next iteration to find the new best three solutions and repeat this process until the stopping condition is satisfied.

Algorithm 1 presents the GWO technique.

**Algorithm 1:** GWO Algorithm

1: Initialize the grey wolf population *X_i_* (*i* = 1, 2, 3,…,*n*) and *t* = 1.
2: Initialize *C*, *a*, and *A* using Equations (7) and (8).
3: Calculate the fitness of each search agent.
4: Put the best search agent as *X*_α_,
the second best search agent as *X*_β_ and
the third best search agent as *X*_δ_.
5: **while** (*t* < Max number of iterations)
6: **for** each search agent
7: Modify the current search position using Equation (11).
8: **end for**
9: Update *a*, *A*, and *C*.
10: Calculate the fitness of all search agents.
11: Modify *X*_α_, *X*_β_ and *X*_δ_.
12: *t* = *t* + 1.
13: **end while**
14: **return** *X*_α_.

## Grey Wolf Reconstruction Based Algorithm

In this section, the proposed GWRA is described. GWRA can be used by the base station to reconstruct the sensors readings again. GWRA algorithm is inspired from the GWO algorithm and the reversible GA. GWRA has two forward steps (*GA forward* and *GWO forward)* and one backward step. In the first iteration, GWRA starts like any GA by initializing the support-set *R* with *q* elements using MF detection (*GA forward step*). GWRA increases the search space (search set *C*) by selecting extra *K* elements depending on GWO algorithm (*GWO forward step*). Then, GWRA solves the LS equation to select the best *k* elements from *q* + *K* elements (*backward step*).

**Figure 2:**
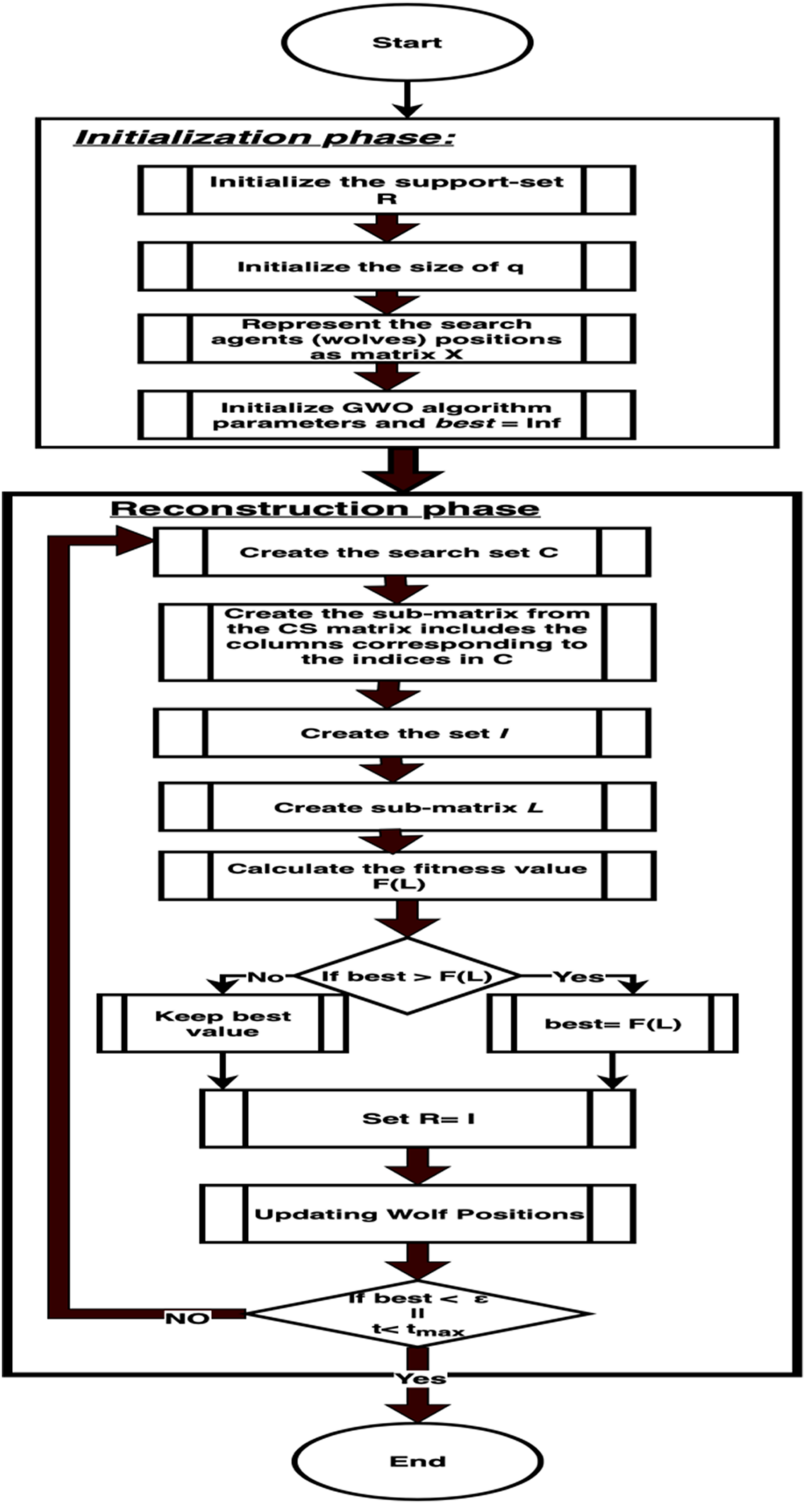
GWRA algorithm flow chart.

At the end of this iteration, GWRA updates the support-set *R* with these *K* elements. From the second iteration, GWRA depends only on GWO forward step to select new *k* elements and add them to *C*, i.e., *C* has 2 * *K* elements in search space in each iteration to select the best *k* elements till it reaches the maximum number of iterations. Flow chart of GWRA is shown below in Fig. 2.

The difference between GWRA and the other reversible GA like CoSaMP (Needell & Tropp, 2009) and SP (Dai & Milenkovic, 2009) algorithm is that in each iteration, GWRA uses the strength of GWO algorithm to find the best *k* according to their fitness values that leads the search toward the optimal solution. GWRA consists of two phases: initialization and reconstruction, as described below.

### Initialization phase

Grey wolf reconstruction algorithm performs the following initialization in this phase:

[1]. Initialize the support-set *R* with indices of φ^*T*^ columns that corresponds to the largest *q* magnitude components in *H*, where *H* = φ^*T*^*y*.
[2]. Initialize the size of *q* to *M*/2 − *K* depending on the fact “CS reconstruction problem can be resolved if the sparsity level *K* ≤ *M*/2” [2]. Initializations [1] and [2] will be executed only once at the beginning of the GWRA.
[3]. Represent the search agents (wolves) positions as matrix *X*_*i × j*_, where *i* = number of wolves and *j* = *K*. Each value of this matrix is a randomly selected integer [1, *N*], where *N* denotes the count of columns in φ, where each number represents an index of a column in φ without duplication.
[4]. Initialize *X*_α_, *X*_β_ and *X*_δ_ as vector 1 × *K* all of its components equal to 0s.
[5]. Initialize best = Sec_best_ = thir_best_ = infinity.
[6]. Initialize outer-loop iteration *t* = 1.
[7]. Initialize the stopping threshold ε = 10^−5^.
[8]. Initialize the estimated solution *x*′ = ø.

### Reconstruction phase

The details of the reconstruction phase are described as given below:

[1]. For each row *i* in matrix *X* do the following:
  - Create the search set *C*, where *C* = *R* ∪ {Row #*i* of *X*_*i × j*_}.
  - Create the sub-matrix φ_c_ from the CS matrix φ. φ_c_ includes the columns corresponding to the indices in *C*.
  - Create the set *I* as the *K* indices in *C* that have largest amplitude components of φ_c_^†^*y*.
  - Create sub-matrix *L* = φ_I_, the columns of matrix φ that corresponds to indices in set *I* (*backward step*).
  - Calculate the fitness value *f*(*L*), GWRA uses the same fitness function in Du, Cheng & Chen (2014) which can be expressed as follows:
    (12) $$f\left( L \right) = {\left\| {L{L^\dagger }\left. {y - y} \right\|} \right._{2}}$$
  - If best > *f*(*L*), then best = *f*(*L*) and *X*_α_ = *I*.
  - Otherwise if best < *f*(*L*) and Sec_best_ > *f*(*L*), then Sec_best_ = *f*(*L*) and *X*_β_ = *I*.
  - Otherwise if best < *f*(*L*), Sec_best_ < *f*(*L*) and thir_best_ > *f*(*L*), then thir_best_ = *f*(*L*) and *X*_δ_ = *I*.
  - Set *R* = *I*.
[2]. **Updating wolves position:** This step updates each search agent’s position according to Eq. (11). The matrix *X* is updated according to the new position of *X*_α_, *X*_β_ and *X*_δ_.
[3]. In order to keep the values of Matrix *X* as integer values between [1, *N*], we modified Eq. (11) as follows:
  (13) $X\left({t + 1} \right) = {\rm{Ceil}}\left({{\rm{Mod}}\left({{{{X_1} + {X_2} + {X_3}} \over 3},N} \right)} \right)$
[4]. Check if *t* (the number of iterations) is less than the maximum count of iterations *t*_max_ or best > ε where ε = 10^−5^, then *t* = *t* + 1 and go to [1] else stop and return *x*′ where *x*′_*I*_ = *L*^†^*y* and *x*′_*S*−*I*_ = 0 where *S* = [1, 2… *N*].

Algorithm 2 presents the GWRA algorithm.

**Algorithm 2:** GWRA

**1: Input**: CS matrix φ_M × N_, measurement vector y and sparsity level **K.**
**2: Output**: estimated solution set x′**:**
**Initialization phase:**
3: R ≜ {indices of the q largest magnitude entries in φ^T^y}.
4: Initialize the grey wolf population matrix X_i × K_ with random integers between [1, N].
5: X_α_ = zeros (1, K), X_β_ = zeros (1, K), X_δ_ = zeros (1, K).
6: best = Sec_best_ = thir_best_ = ∞.
7: x′ = ø, ε = 10^−5^ and t = 1.
**Reconstruction phase:**
8: **while** (t < t_max_||best > ε)
9: **for** each row i of the matrix X_i × K_ **do**
10: C = Union(R, Row #i of X_i × j_)
11: I ≜ {indices of the K largest magnitude entries in φ_c_^†^ y}.
12: L = φ_I_.
13: Calculate the fitness value f(L) using Equation 12.
14. **If**(best > f(L)), **then**
15: X_α_ = I,
16: **Else If** (best < f(L) && Sec_best_ > f(L)), **then**
17: Sec_best_ = f(L) and X_β_ = I.
18: **Else If** (best < f(L) && Sec_best_ < f(L) && thir_best_ > f(L)), **then**
19: thir_best_ = f(L) and X_δ_ = I.
20: **End If**
21: **Set** R = I.
22: **end for**
23: Wolf positions updating step:
24: Update a, A, and C
25: **for** each search agent
26: Update the position of the current search agent by Equation (13).
27: **end for**
28: t = t + 1
29: **End while**
30: **return** x′ where x′_I_ = L^†^y and x′_S−I_ = L^†^y where S = [1, 2… N].

### Example scenario

For clarification, we illustrate the actions of GWRA using the following example:

**Input**: matrix ϕ_6 × 10_ (*M* = 6 and *N* = 10) with elements generated from uniform distribution, *y* = ϕ*x* ∈ *R*^6^ is the compressed samples and the sparsity level *K* = 2.

**Output**: estimated signal *x*′.

$$\eqalign{& {{\rm\phi} _{6\,\, \times \,\,10}} = \cr & \left( {\matrix{ {0.023} & {0.275} & {0.364} & {0.249} & {0.150} & {0.983} & {0.525} & {0.9753} & {0.824} & {0.075} \cr {0.489} & {0.847} & {0.207} & {0.287} & {0.561} & {0.412} & {0.456} & {0.972} & {0.360} & {0.592} \cr {0.945} & {0.804} & {0.847} & {0.155} & {0.271} & {0.502} & {0.194} & {0.306} & {0.541} & {0.970} \cr {0.967} & {0.062} & {0.979} & {0.609} & {0.606} & {0.266} & {0.214} & {0.739} & {0.753} & {0.573} \cr {0.853} & {0.594} & {0.374} & {0.156} & {0.973} & {0.260} & {0.713} & {0.773} & {0.850} & {0.974} \cr {0.181} & {0.483} & {0.452} & {0.460} & {0.357} & {0.339} & {0.549} & {0.538} & {0.911} & {0.598} \cr } } \right), \cr & y = \left[ {\matrix{{0.106} \cr {0.560} \cr {0.560} \cr {0.784} \cr {0.973} \cr {0.303} \cr } } \right],\ x = \left[ {\matrix{{0.408} \cr 0 \cr 0 \cr 0 \cr {0.641} \cr 0 \cr 0 \cr 0 \cr 0 \cr 0 \cr } } \right] \cr}$$

#### Initialization phase execution

1. Support-set *R* = {10}, the indices of columns of ϕ that correspond to the largest *q*(= 1) amplitude components in *H* = φ^*T*^*y*, where $q = M/2 - K = 1$.
  $$\hskip -1.2pc\eqalign{ & H = \left( {\matrix{ {0.023} & {0.275} & {0.364} & {0.249} & {0.150} & {0.983} & {0.525} & {0.9753} & {0.824} & {{0.075}} \cr  {0.489} & {0.847} & {0.207} & {0.287} & {0.561} & {0.412} & {0.456} & {0.972} & {0.360} & {{0.592}} \cr  {0.945} & {0.804} & {0.847} & {0.155} & {0.271} & {0.502} & {0.194} & {0.306} & {0.541} & {{0.970}} \cr  {0.967} & {0.062} & {0.979} & {0.609} & {0.606} & {0.266} & {0.214} & {0.739} & {0.753} & {{0.573}} \cr  {0.853} & {0.594} & {0.374} & {0.156} & {0.973} & {0.260} & {0.713} & {0.773} & {0.850} & {{0.974}} \cr  {0.181} & {0.483} & {0.452} & {0.460} & {0.357} & {0.339} & {0.549} & {0.538} & {0.911} & {{0.598}} \cr  } } \right)^T \cr  & \left[ {\matrix{ {0.106} \cr  {0.560} \cr  {0.560} \cr  {0.784} \cr  {0.973} \cr  {0.303} \cr  } } \right] = \left[ {\matrix{ {2.450} \cr  {1.729} \cr  {1.899} \cr  {1.044} \cr  {2.014} \cr  {1.181} \cr  {1.450} \cr  {2.316} \cr  {2.288} \cr  {{2.464}} \cr  } } \right] \cr} $$
2. Matrix *X*_*i* × *K*_, where *i* = number of search agents (= 5) and *K* = 2, will be initialized as follows:
  $${X_{5\, \times \,2}} = \left( {\matrix{ 5 & 7 \cr 8 & 6 \cr 9 & 2 \cr 2 & 4 \cr 8 & 2 \cr } } \right)$$
3. Initialize *X*_α_, *X*_β_ and *X*_δ_ as *X*_α_ = [0 0], *X*_β_ = [0 0] and *X*_δ_ = [0 0].
  best = Sec_best_ = thir_best_ = ∞. Number of the outer-loop iteration is initialized to *t* = 1 and the estimated solution *x*′ = ø.

#### Reconstruction phase execution

1. For each row *i* in the matrix do: when *i* = 1
  - $$C = R \cup \{ {\rm{row }}\,1\,{\rm{ of \ }}{X_{5 \times 2}}\}  = \{ 10,5,7\} ,$$
  - Create the sub-matrix ϕ_*c*_ by selecting the columns from ϕ which correspond to the indices in *C*.
    $${{\phi} _{c\, = \,\left\{ {5,\,7,\,10} \right\}}} = \left({\matrix{ {0.150} & {0.525} & {0.075} \cr {0.561} & {0.456} & {0.592} \cr {0.271} & {0.194} & {0.970} \cr {0.606} & {0.214} & {0.573} \cr {0.973} & {0.713} & {0.974} \cr {0.357} & {0.549} & {0.598} \cr } } \right)$$
  - The set *I* will be created as the indices of the largest *K*(= 2) amplitude components in ϕ_*c*_^†^*y*:
    $${\phi _{c\, = \,\left\{ {5,\,7,\,10} \right\}}}^\dagger y = \left( {\matrix{ {0.150} & {0.525} & {0.075}  \cr {0.561} & {0.456} & {0.592}  \cr {0.271} & {0.194} & {0.970}  \cr {0.606} & {0.214} & {0.573}  \cr {0.973} & {0.713} & {0.974}  \cr {0.357} & {0.549} & {0.598}  \cr } } \right)\dagger \left[ {\matrix{ {0.106}  \cr {0.560}  \cr {0.560}  \cr {0.784}  \cr {0.973}  \cr {0.303}  \cr } } \right] = \left[ {\matrix{ {0.927}  \cr {0.300}  \cr {0.338}  \cr } } \right]$$
    i.e., *I* = {5, 10}. And then we create the sub-matrix $L = \,{{\phi} _I} = \left( {\matrix{ {{0.150}} & {{0.075}} \cr  {{0.561}} & {{0.592}} \cr  {{0.271}} & {{0.970}} \cr  {{0.606}} & {{0.573}} \cr  {{0.973}} & {{0.974}} \cr  {{0.357}} & {{0.598}} \cr  } } \right)$
  - Using Eq. (12), the fitness value *f*(*L*) of the sub-matrix will be 0.233.
  - Since best > *f*(*L*), best = 0.233, *X*_α_ = {5, 10}.
2. Repeating the same steps for all rows (*i* = 2, 3, 4, 5) of *X*, we will have best = 0.233, *X*_α_ = *I* = {5, 10}. *R* will be updated as *R* = *I* = {5, 10}.
3. Using Eq. (13), the updated position matrix *X* will be:
  ${X_{5\,\, \times \,\,2}} = \left({\matrix{ 1 & 8 \cr 6 & 3 \cr 4 & 8 \cr 7 & 9 \cr 7 & 6 \cr } } \right)$
4. Since the stop criteria are not satisfied, the iteration number will be updated *t* = *t* + 1 and execute **Reconstruction phase** as follows:
  For each row *i* in the matrix do: (*when i* = 1)
  - *C* = *R* ∪ {row 1 in *X*} = {10, 5, 1, 8},
  - Create the sub-matrix ϕ_*c*_ by selecting the columns from matrix ϕ that correspond to indices in *C*.
    $${{\phi} _{c\, = \,\left\{ {1,\,5,\,8,\,10} \right\}}} = \left({\matrix{ {0.023} & {0.150} & {0.9753} & {0.075} \cr {0.489} & {0.561} & {0.972} & {0.592} \cr {0.945} & {0.271} & {0.306} & {0.970} \cr {0.967} & {0.606} & {0.739} & {0.573} \cr {0.853} & {0.973} & {0.773} & {0.974} \cr {0.181} & {0.357} & {0.538} & {0.598} \cr } } \right)$$
  - Create the set *I* as the indices of the largest *K* amplitude components in ${{\phi} _c}^\dagger y$:
    $${{\phi} _c}^\dagger y = \left( {\matrix{ {0.023} & {0.150} & {0.9753} & {0.075} \cr  {0.489} & {0.561} & {0.972} & {0.592} \cr  {0.945} & {0.271} & {0.306} & {0.970} \cr  {0.967} & {0.606} & {0.739} & {0.573} \cr  {0.853} & {0.973} & {0.773} & {0.974} \cr  {0.181} & {0.357} & {0.538} & {0.598} \cr  } } \right)^\dagger \left[ {\matrix{ {0.106} \cr  {0.560} \cr  {0.560} \cr  {0.784} \cr  {0.973} \cr  {0.303} \cr  } } \right] = \left[ {\matrix{ {{0.408}} \cr  {{0.641}} \cr  {0.2338} \cr  {0.2254} \cr  {0.3215} \cr  } } \right]$$
    i.e., *I* = {1, 5}.
  - The sub-matrix *L* will be: $L = \,{{\rm\phi} _I} = \left( {\matrix{ {0.023} & {0.150} \cr  {0.489} & {0.561} \cr  {0.945} & {0.271} \cr  {0.967} & {0.606} \cr  {0.853} & {0.973} \cr  {0.181} & {0.357} \cr  } } \right)$
  - Using Eq. (12), the fitness value *f*(*L*) of the sub-matrix *L* will be 10^−16^.
  - Since best > *f*(*L*), then best = 10^−16^, *X*_α_ = {1, 5}.
5. Repeating the same steps for every row of X (*i* = 2, 3, 4, 5) in the wolf position matrix *X*, we will have best = 10^−16^, *X*_α_ = {1, 5}, and updated *R* = I = {1, 5}.
6. Update each search agent’s position (matrix *X*) according to Eq. (13):
  $${X_{5\,\, \times \,2}} = \left({\matrix{ 2 & 7 \cr 5 & 3 \cr 4 & 5 \cr 1 & 9 \cr 5 & 6 \cr } } \right)$$
7. According to the stop criteria best <10^−5^, stops and calculates *x*′ as following:
  $${x'_{I = \,\left\{ {1,\,5} \right\}}} = {L^\dagger }y = \left( {\matrix{ {0.023} & {0.150}  \cr {0.489} & {0.561}  \cr {0.945} & {0.271}  \cr {0.967} & {0.606}  \cr {0.853} & {0.973}  \cr {0.181} & {0.357}  \cr } } \right)\left[ {\matrix{ {0.106}  \cr {0.560}  \cr {0.560}  \cr {0.784}  \cr {0.973}  \cr {0.303}  \cr } } \right] = \left[ {\matrix{ {0.408}  \cr {0.641}  \cr } } \right]{\rm{and\,then\,set}}\,{x'_{S - I\, = \,\left\{ {2,\,3,\,4,\,6,\,7,\,8,\,9,\,10} \right\}}} = 0$$

Then, the estimated signal *x*′ will be as follows: $x' = \left[ {\matrix{{0.408}\cr0\cr0\cr0\cr{0.641}\cr0\cr0\cr0\cr0\cr0\cr}}\right]$ which is equals to *x*.

Therefore, GWRA succeeds to reconstruct the original data without any errors.

## Simulation Results

In this section, the MATLAB environment is used for performing all simulations and the reconstruction is investigated by Gaussian matrix Φ, of size *M* × *N*, where *M* = 128 and *N* = 256. Two types of data are used to evaluate the reconstruction performance of the proposed algorithm: computer generated data and real data set. In the first type, we used data generated from Uniform and Gaussian distribution as an example to evaluate the proposed algorithm. The whole process is repeated over 500 times and then averaged on randomly generated *K* sparse samples. The performance evaluation of GWRA and its comparison with the baseline algorithms such as CoSaMP (Needell & Tropp, 2009), OMP (Tropp & Gilbert, 2007), SP (Dai & Milenkovic, 2009), FBP (Burak & Erdogan, 2013), BA (Bao et al., 2018) and PSO (Du, Cheng & Liu, 2013) in terms of both average normalized mean squared error (ANMSE) and mean absolute percentage error (MAPE) is given below. The setting of used Parameters is shown in Table 3.

**Table 3:** Parameters setting.

Parameter	Value
Signal length (*N*)	256
Measurement vector length (*M*)	128
CS matrix (Φ)	128 × 256
Sparse level (*K*)	From 5 to 60 with five increment
search agents matrix *X_i_*_× *j*_	*i* = 100, *j* = *K*
Compression ratio	70%, 60%, 50%, 40% and 30%

**Performance Metrics***:* The GWRA algorithm reconstruction’s performance is compared with different reconstruction algorithms in terms of the following performance metrics:

1. Average normalized mean squared error: the average ratio ${}^{x-{x}'}\!\!/{{{x}_{2}}}\;$ defines the ANMSE, where *x* represents the initial reading and *x*′ represents the reconstructed one.
2. Mean absolute percentage error: the ratio ${\raise0.7ex\hbox{${\sum {\left| {{{x - x'} \over x}} \right|} }$} \!\mathord{\left/ {\vphantom {{\sum {\left| {{{x - x'} \over x}} \right|} } n}}\right.}\!\lower0.7ex\hbox{$n$}}$ defines the MAPE.

### Average normalized mean squared error evaluation

The GWRA algorithm is evaluated in terms of ANMSE and the result is compared with the existing algorithms.

**Figure 3:**
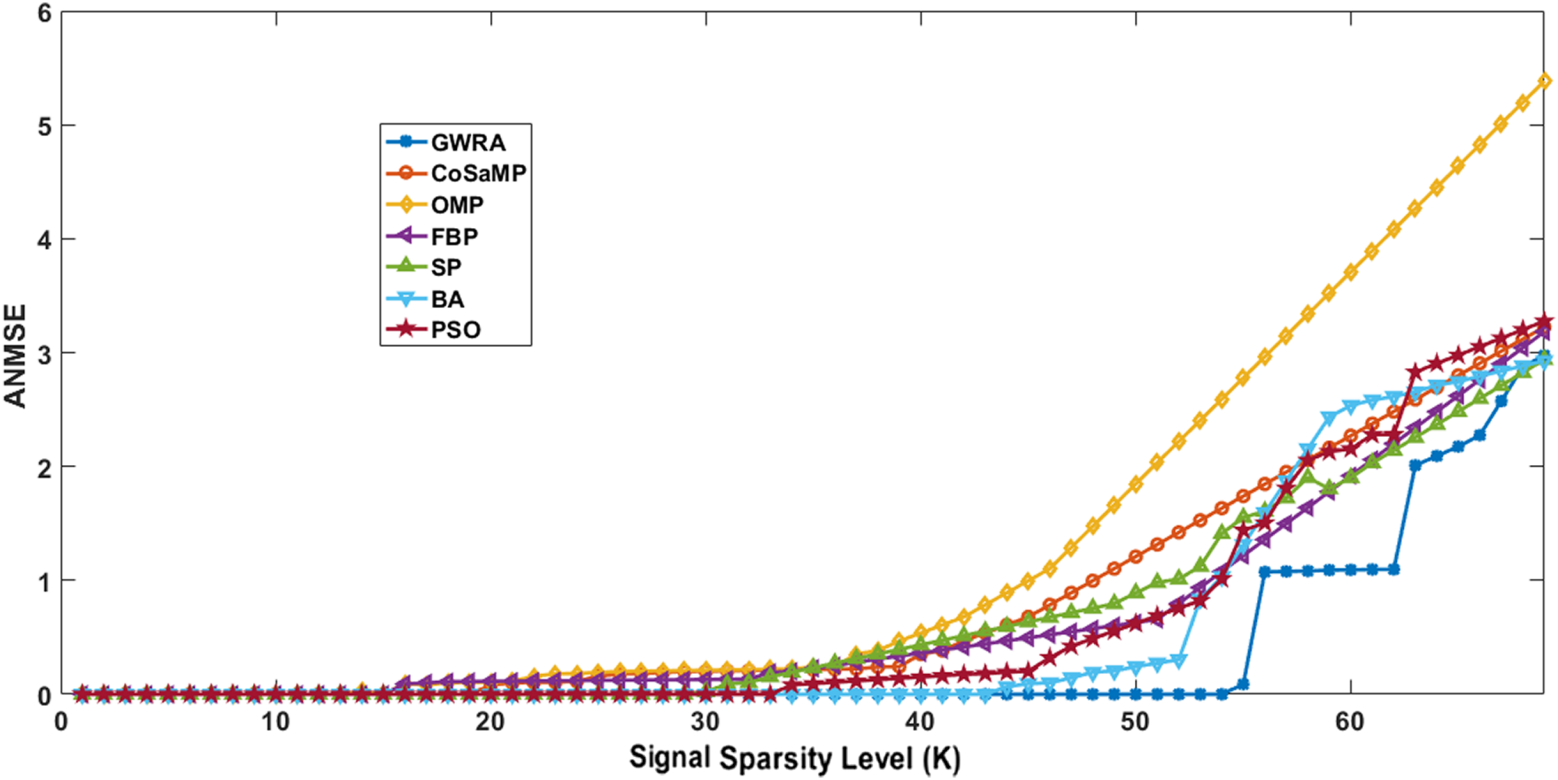
ANMSE in GWRA, CoSaMP, OMP, FBP, SP, BA and PSO algorithms over generated Gaussian sparse vector.

Figure 3 illustrates the results of ANMSE evaluation in which Gaussian distribution is used to generate the non-zero entries of the sparse signal. The results prove that GWRA algorithm provides reduced ANMSE than CoSaMP, OMP, FBP, BA, PSO and SP. Also, the ANMSE of GWRA starts to increase only when *K* > 57 while it increased when *K* > 22, *K* ≥ 19, *K* ≥ 26, *K* ≥ 33, *K* ≥ 46, *K* ≥ 38 for CoSaMP, OMP, FBP, SP, BA, PSO, respectively as shown in Fig. 3. This is because GWRA applies the grey wolves’ behavior to hunt the prey (*k* elements) inside search space (CS matrix) according to their fitness values (the best fitness values). Then, in each iteration, the support-set will be updated with the best *k* elements, i.e., GWRA has the best-estimated solution till it reaches the optimal one.

**Figure 4:**
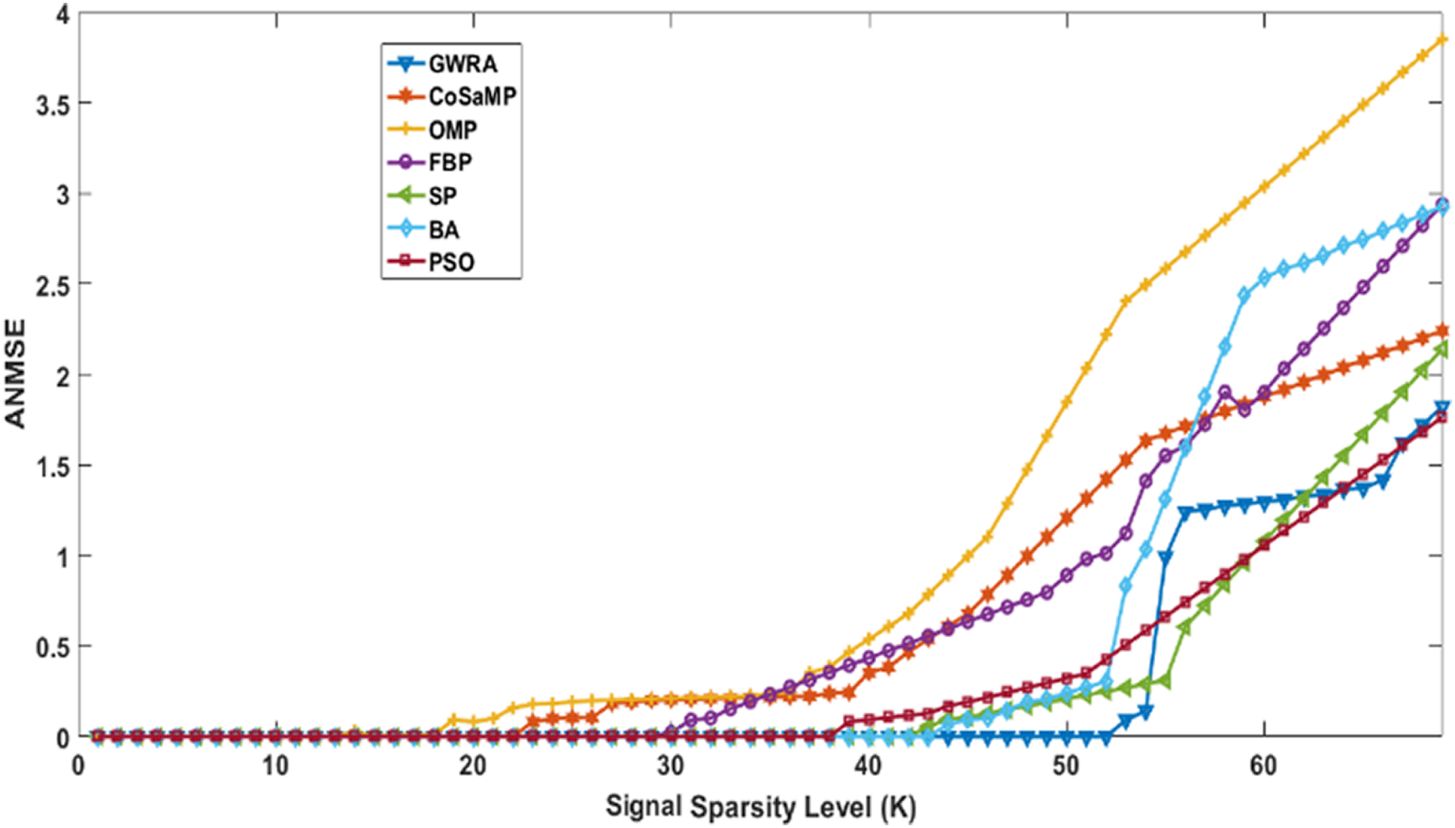
ANMSE in GWRA, CoSaMP, OMP, FBP, SP, BA and PSO algorithms over generated Uniform sparse vector.

Figure 4 illustrates the results of ANMSE evaluation in which Uniform distribution is used to generate the non-zero entries of the sparse signal. The results prove that GWRA algorithm still gives the lowest ANMSE value than CoSaMP, FBP, OMP, SP, BA, PSO as *K* > 53, *K* ≥ 25, *K* > 20, *K* > 26, *K* > 33, *K* ≥ 45, *K* > 37, respectively, because what any GA does in one round, GWRA does it for each search agent and then it selects the best one in every iteration to converge at the optimal solution.

**Figure 5:**
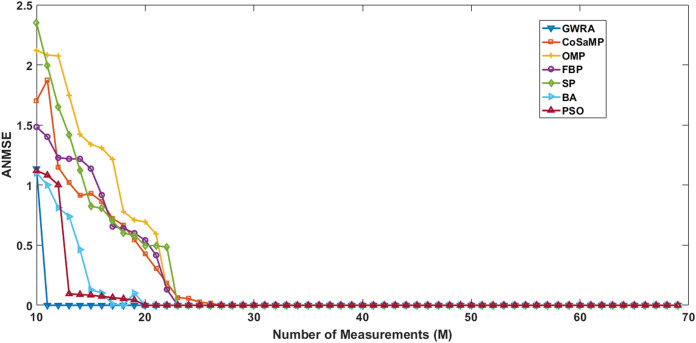
ANMSE in GWRA, CoSaMP, OMP, FBP, SP, BA and PSO algorithms over generated Gaussian matrix with different lengths of *M*.

In the second test, we measure the reconstruction performance of GWRA as a function in terms of the length of measurement-vector and then compared the results using CoSaMP, FBP, SP, BA, OMP, PSO. The sparse signals are generated using Gaussian distribution having length *N* = 120, *M* values varying from 10 to 60 with increment of 1. Illustration of the reconstruction performance of GWRA, CoSaMP, OMP, FBP, SP, BA and PSO with different measurement vector length, *M* is given in Fig. 5. From the figure, we observe that GWRA algorithm still gives the lowest ANMSE results compared to the others.

**Figure 6:**
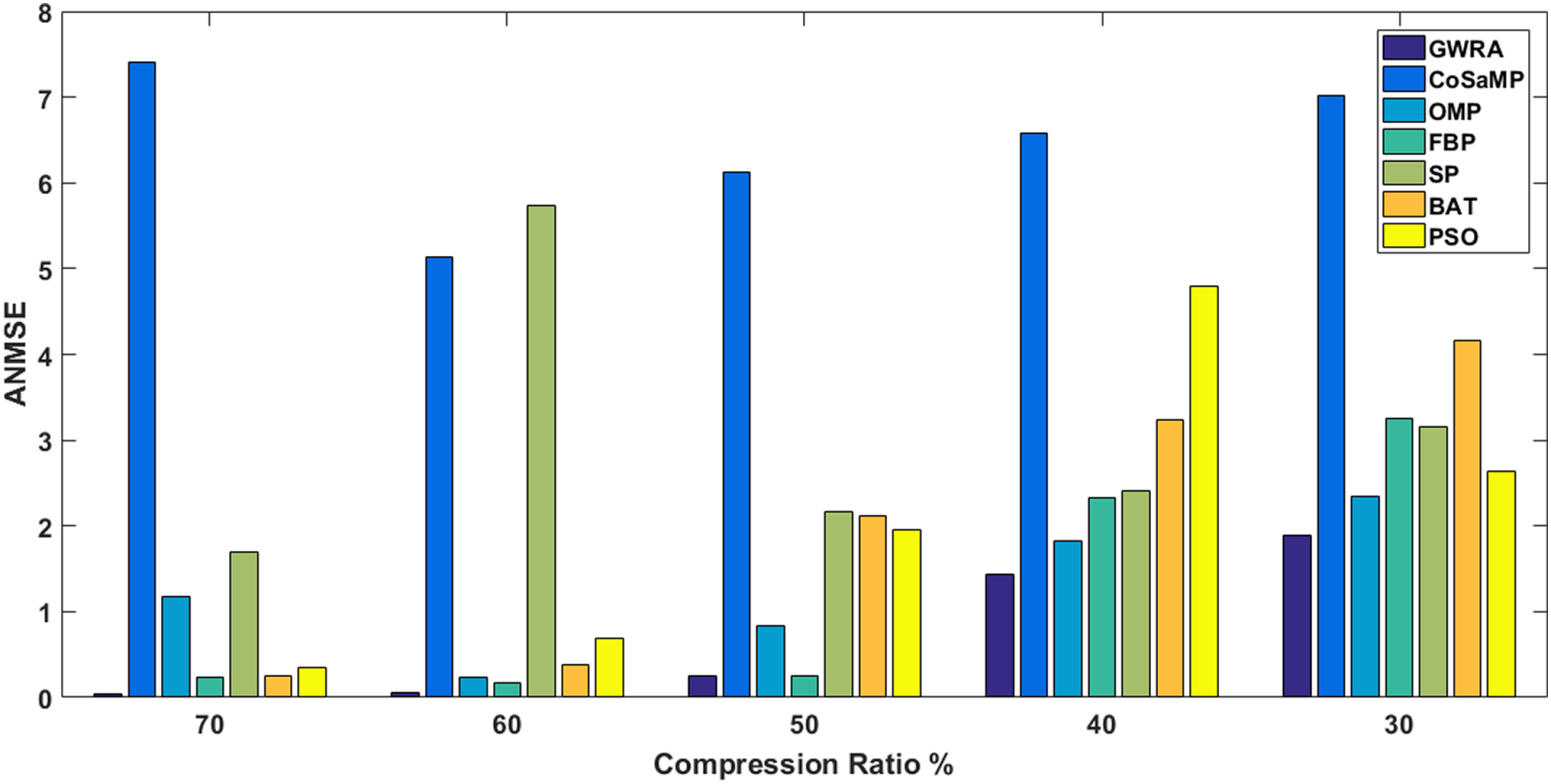
ANMSE in GWRA, CoSaMP, OMP, FBP, SP, BA and PSO algorithms for different compression ratios.

In the third test, reconstruction performance of GWRA is measured in terms of ANMSE as a function of compression ratio over Uniform and Gaussian sparse vectors as shown in Fig. 6 and Table 4, respectively. In this test, we have *N* = 256 and the different compression ratios are 70%, 60%, 50%, 40% and 30% where *K* = *M*/2. Figure 6 shows the ANMSE for GWRA, CoSaMP, OMP, FBP, SP, BA and PSO for different compression ratios. From Fig. 6, we can conclude that GWRA algorithm achieves the best reconstruction performance with different compression ratio. The same performance can be noted from Table 4, where GWRA achieves the minimum reconstruction error in comparison to the other algorithms for different compression ratio values.

**Table 4:** ANMSE for different compression ratios over generated Gaussian sparse vector.

Compression ratio (%)	GWRA	COSAMP	OMP	FBP	SP	BA	PSO
70	3.10710*e*^−29^	5.135	0.228	0.1717	1.699	0.245	0.354
60	0.0583	6.1224	0.828	0.2412	2.164	0.389	0.687
50	0.2515	6.575	1.125	0.2572	2.415	2.124	1.953
40	1.4313	7.025	1.820	2.3341	3.156	3.245	2.644
30	1.894	7.4220	2.348	3.2498	5.125	4.165	4.789

### Mean absolute percentage error evaluation

**Figure 7:**
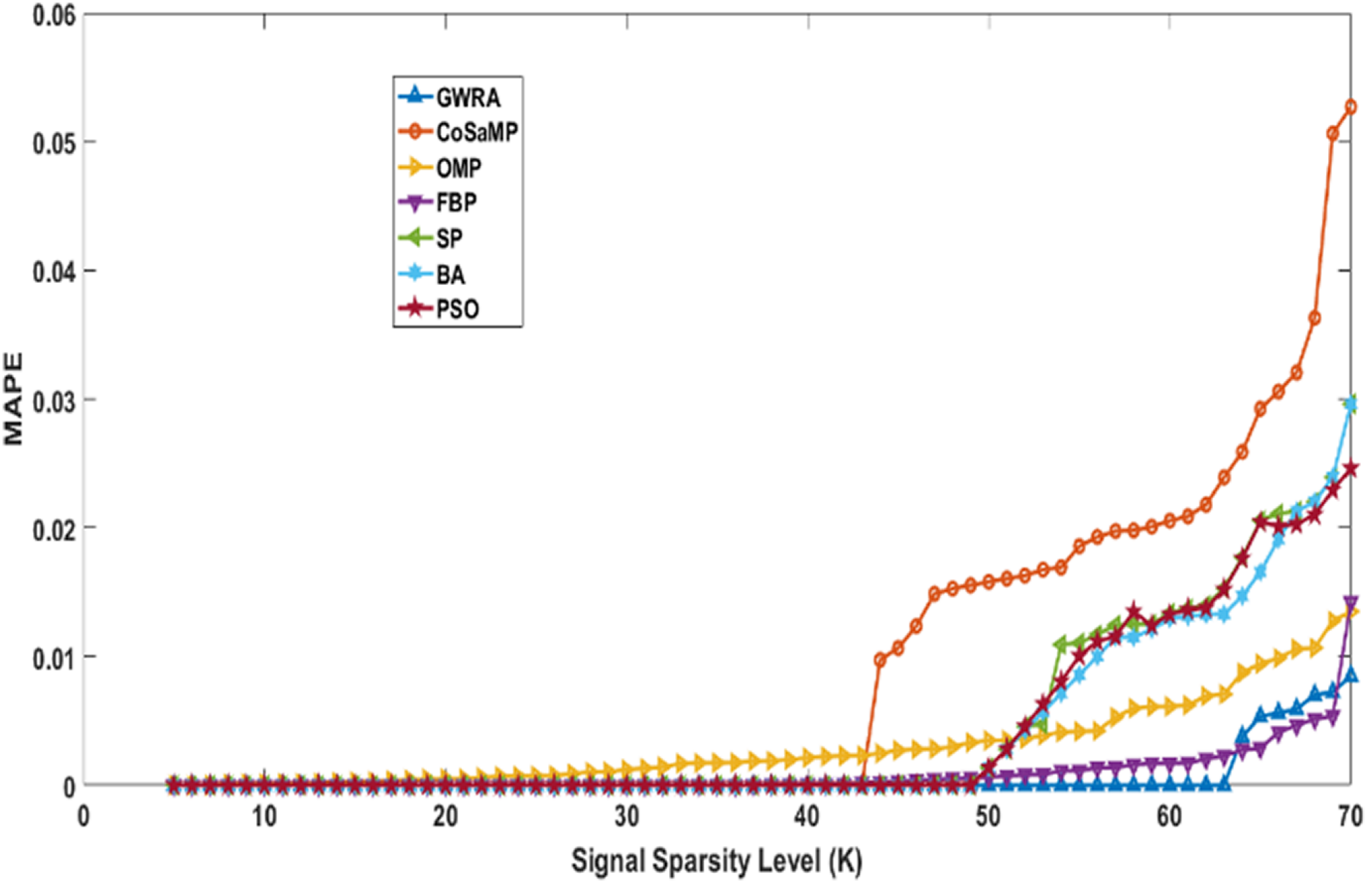
MAPE over sparsity for Uniform sparse vector in GWRA, CoSaMP, OMP, FBP, SP, BA and PSO.

In the fourth test, we measure the reconstruction performance of GWRA in terms of MAPE and the result is compared with other algorithms. Figure 7 shows MAPE results for GWRA, CoSaMP, OMP, FBP, SP, BA, PSO algorithms and it is clear that GWRA exceeds the reconstruction performance of others in terms of reducing the MAPE, because GWRA integrates the advantages of both greedy as well as the GWO algorithm to achieve the best result.

### Case study

Here, we demonstrate the effectiveness of the GWRA algorithm introduced in this paper in reducing ANMSE and MAPE. For this purpose, the proposed algorithm is applied to reconstruct real weather dataset (Ali, Gao & Mileo, 2018). This dataset contains weather observations of Aarhus city, Denmark obtained during February–June 2014 and also August–September 2014.

**Figure 8:**
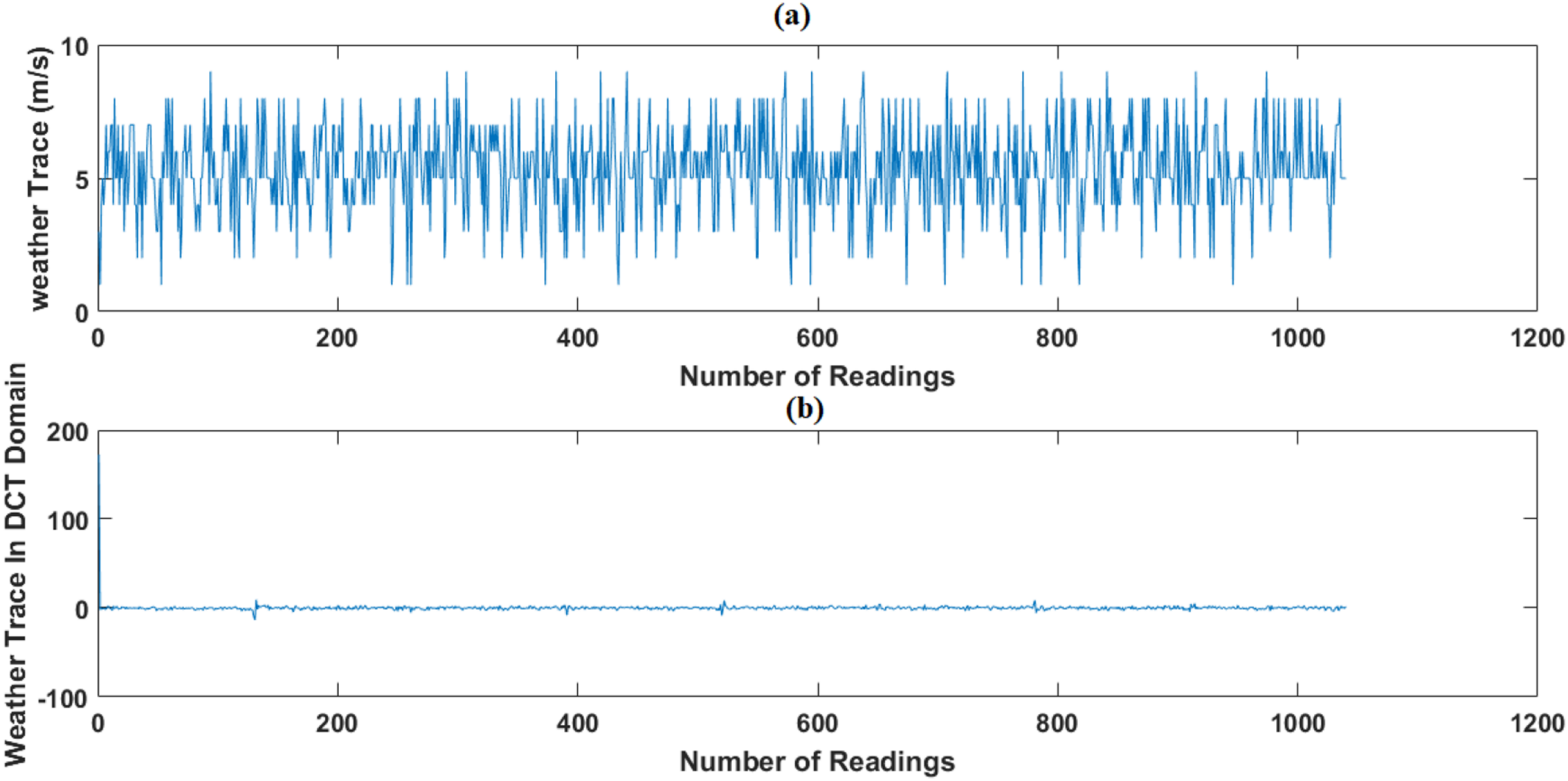
Weather trace in DCT Domain: (A) the original data and (B) the sparse signal representation.

**Figure 9:**
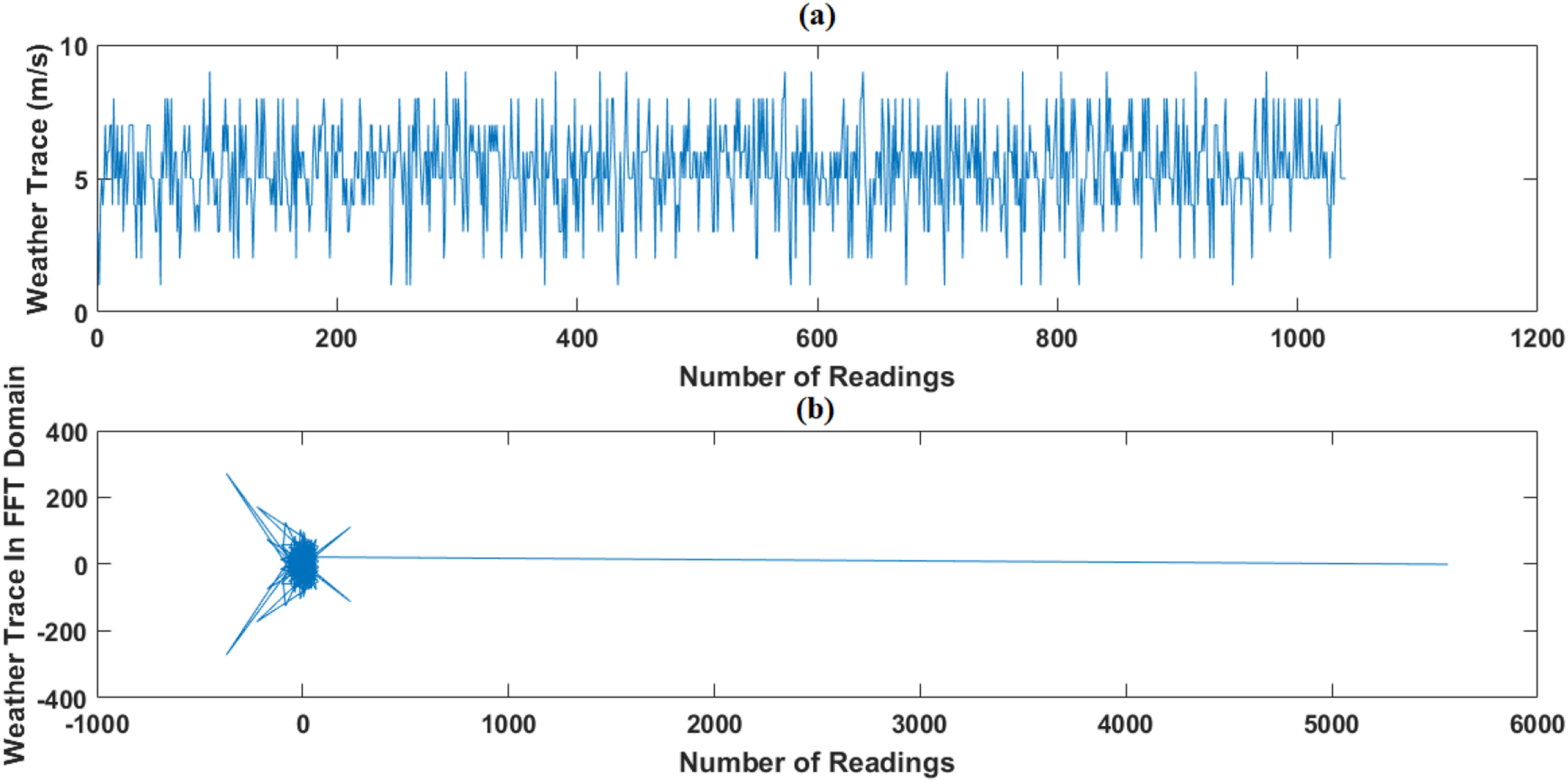
Weather trace in FFT domain: (A) the original data and (B) the sparse signal representation.

In this test, we use the weather dataset of February 2014 period as original data. Using CS, February dataset is compressed, then we apply, evaluate and compare the performance of GWRA, CoSaMP, OMP, FBP, LP (Pant, Lu & Antoniou, 2014) and SP to recover it back. In addition, we use DCT (Duarte-Carvajalino & Sapiro, 2009) and FFT (Canli, Gupta & Khokhar, 2006) as sparse domain, as shown in Figs. 8 and 9.

**Figure 10:**
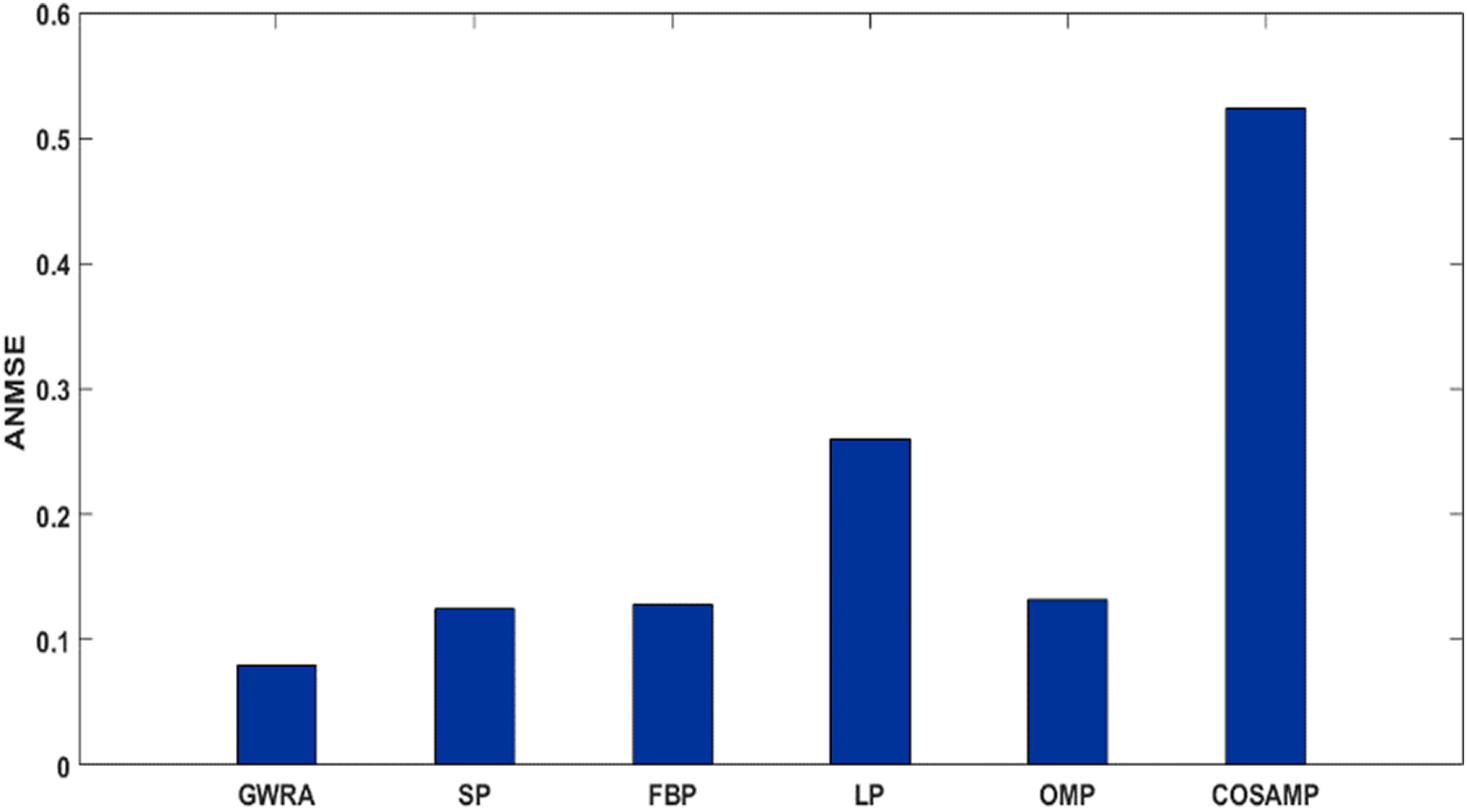
ANMSE in GWRA, SP, FBP, LP, OMP and CoSaMP algorithms using DCT domain (case study).

**Figure 11:**
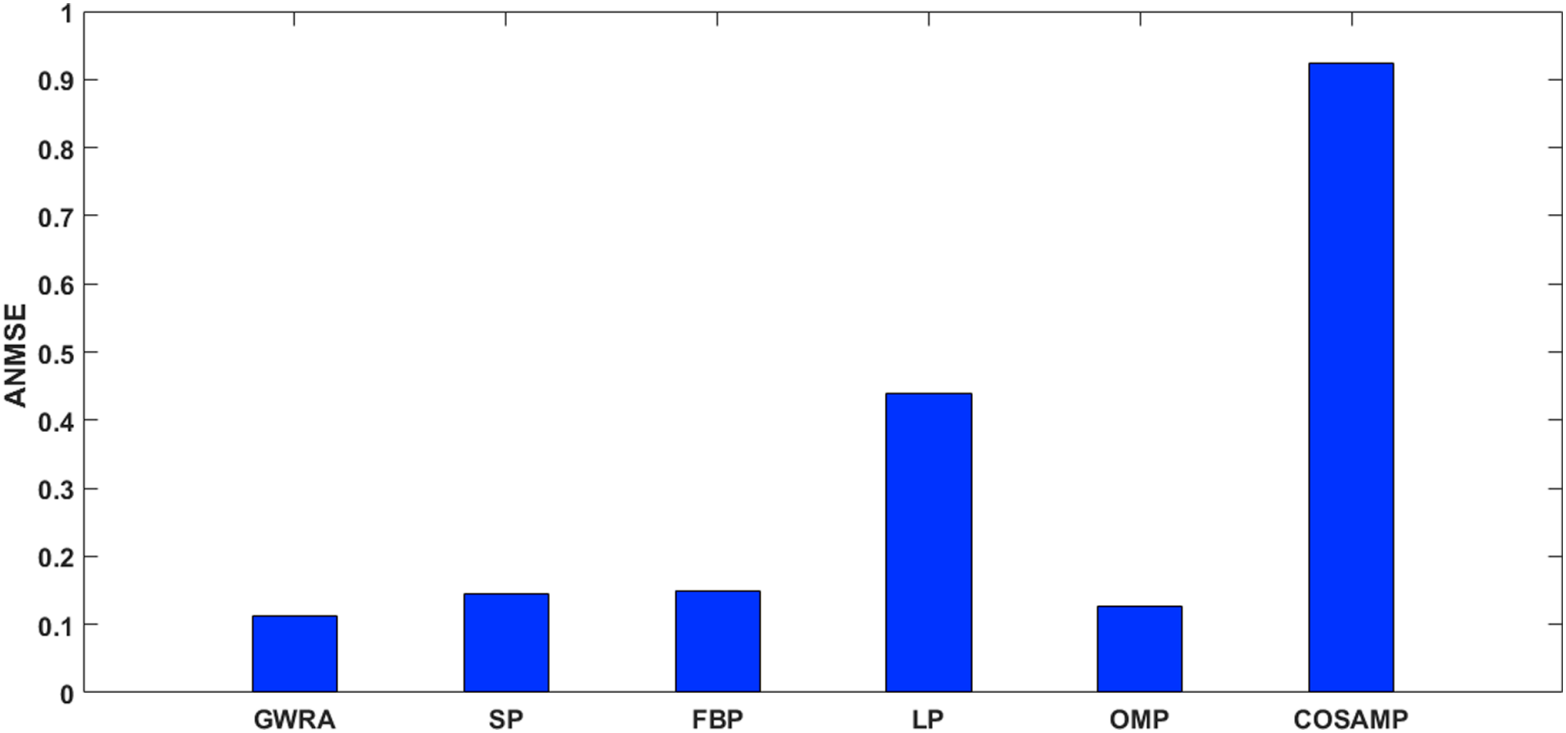
ANMSE in GWRA, SP, FBP, LP, OMP and CoSaMP algorithms using FFT domain (case study).

Figure 10 shows the ANMSE of GWRA, CoSaMP, OMP, FBP, LP and SP using DCT domain. It is clear that GWRA achieves the great performance in reducing ANMSE than other algorithms in case of using DCT as a signal transformer. Figure 11 shows that using FFT domain as signal transformer, the ANMSE of all algorithms increases, but still GWRA provides the best performance.

**Figure 12:**
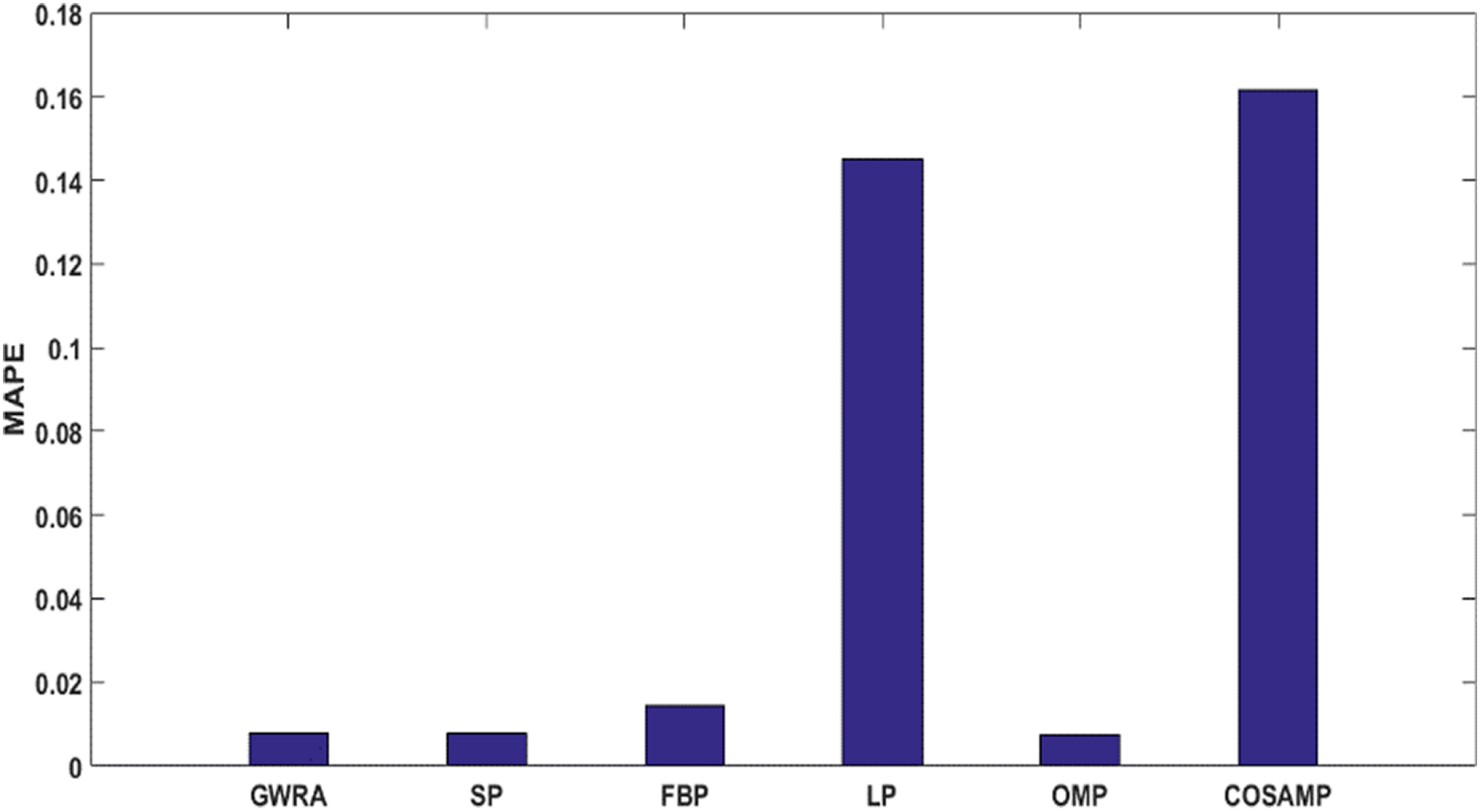
MAPE in GWRA, SP, FBP, LP, OMP and CoSaMP algorithms for weather trace (case study).

As a last test in case study, the performance of GWRA, SP, FBP, LP, OMP and CoSaMP are evaluated in terms of MAPE. It shows that GWRA still succeeds to be superior in the reconstruction performance than the others in terms of reducing MAPE as shown in Fig. 12.

### Complexity analysis

**Figure 13:**
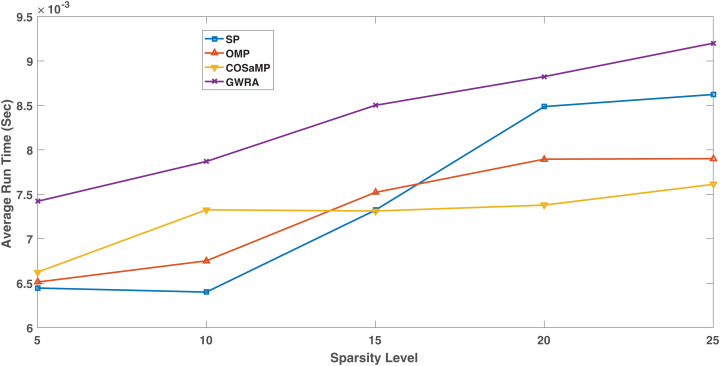
Complexity comparison GWRA, OMP, CoSaMP and SP algorithms.

Figure 13 shows the complexity in the GWRA, OMP, CoSaMP and SP algorithms. It is clear that as swarm algorithm, the complexity of the proposed algorithm is higher than the GA but it is more efficient in data reconstruction. However, the high complexity in GWRA does not represent a problem, since the algorithms will be executed at the BS which has enough hardware capability and not energy constraint.

### Image reconstruction test

**Figure 14:**
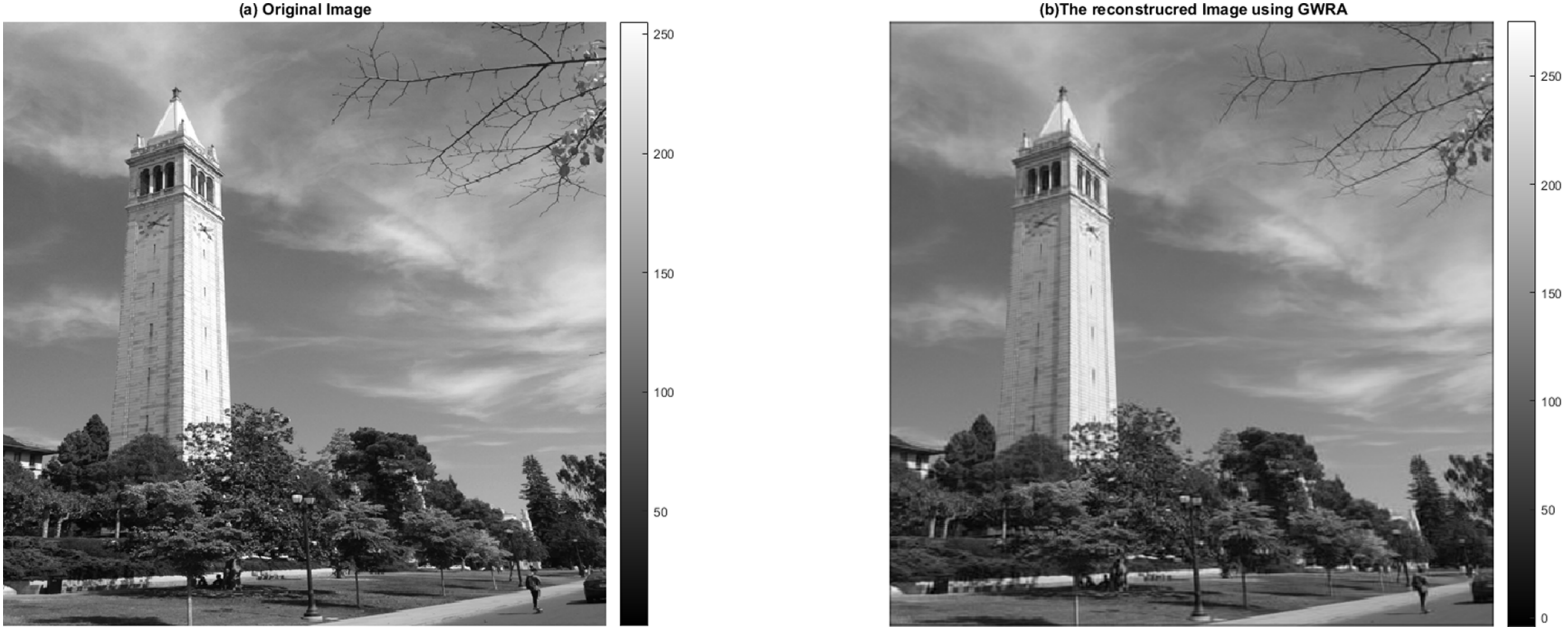
GWRA based image reconstruction test: (A) original image and (B) the reconstructed image.

In this test, we aim to evaluate the reconstruction performance of the GWRA, where it is used to reconstruct 512 × 512 campanile image, which is a typical sight on the Berkeley campus (https://github.com/dfridovi/compressed_sensing) (Fridovich-Keil & Kuo, 2019), as shown in Fig. 14. It can be noted that GWRA efficiently succeeds to reconstruct the test image with small error which proves the efficiency of GWRA.

## Conclusion

In this paper, a novel reconstruction approach for CS signal, based on GWO has been presented which integrates between GA and GWO algorithms to utilize their advantages in fast implementation and finding optimal solutions. In the provided experiments, GWRA exhibited better reconstruction performance for Gaussian and Uniform sparse signals. GWRA achieved overwhelming success over the traditional GA algorithms CoSaMP, OMP, FBP and SP. Also, GWRA provided better reconstruction performance than other swarm algorithms BA and PSO. GWRA successfully reconstructed datasets of weather observations as a case study and it is shown that GWRA succeeded to recover the data correctly with lesser ANMSE and MAPE than compared with existing algorithms. The demonstrated performance prove that GWRA is a promising technique that provides significant reduction in reconstruction errors.

## Supplemental Information

10.7717/peerj-cs.217/supp-1 Supplemental Information 1 OMP Algorithm code by matlab.

## Additional Information and Declarations

### Data Availability

The following information was supplied regarding data availability: MATLAB code is available as a Supplemental File.
